# Dyspnea and a Fistula: A Case Report of an Unusual Cardiac Connection

**DOI:** 10.1155/carm/8082058

**Published:** 2026-05-29

**Authors:** Muhammad Taimur, Khaled M. Harmouch, Mohammed M. Shaheriyar, Yasemin Bahar, Malitha Hettiarachchi, M. Chadi Alraies

**Affiliations:** ^1^ Detroit Medical Center, Wayne State University School of Medicine, Detroit, Michigan, USA, wayne.edu; ^2^ BayCare Health System/St. Anthony’s Hospital, St. Petersburg, Florida, USA; ^3^ Wayne State University, Detroit, Michigan, USA, wayne.edu; ^4^ Cardiovascular Institute, Detroit Medical Center, Detroit, Michigan, USA, dmc.org; ^5^ Michigan State University, East Lansing, Michigan, USA, msu.edu

**Keywords:** angiography, cameral fistulas, cardiac, catheterization

## Abstract

Coronary cameral fistulas are rare abnormal connections between one or more coronary arteries and any chamber of the heart, occurring in less than 1% of the population. Cameral fistulas are classically asymptomatic and an incidental finding. Etiology is most commonly congenital but can also be acquired secondary to cardiac surgery or trauma. Cardiac catheterization with coronary angiography remains the diagnostic modality of choice. We present a case of a symptomatic patient presenting with dyspnea and hypertension diagnosed with an incidental mid‐left anterior descending to right ventricle cameral coronary fistula. We discuss current literature on the management of this anatomic anomaly.

## 1. Introduction

Coronary cameral fistulas (CCF) are rare anomalous connections between one or more coronary arteries and any heart chamber. They are observed in less than 1% of the general population [1]. The most frequently seen variant of CCF cases arises from the right coronary artery (RCA) (55%). However, anomalies arising either from the left coronary system (35%) or bilaterally arising (5%) are also observed in the literature [2]. The right ventricle (RV) (40%) is the most commonly implicated chamber of the heart, followed by the right atrium (RA) (26%) and pulmonary arteries (17%). Connections with other cardiac chambers, including the coronary sinus, left atrium (LA), and left ventricle (LV), are less frequently observed. The etiology of cameral fistulas is usually congenital, but acquired forms have been reported secondary to cardiac surgery, endocarditis, and repeated myocardial biopsies [3].

Cameral fistulas are classically asymptomatic and an incidental finding. Cardiac catheterization with coronary angiography is the diagnostic method of choice [4]. Management of symptomatic cameral fistulas is under ongoing discussion, and surgical repair, catheter closure, and medical management have been explored as potential strategies [2].

We present a case of a symptomatic patient with an incidental mid‐left anterior descending (LAD) to RV cameral coronary fistula and highlight the current literature on this topic.

## 2. Case Presentation

**FIGURE 1:**
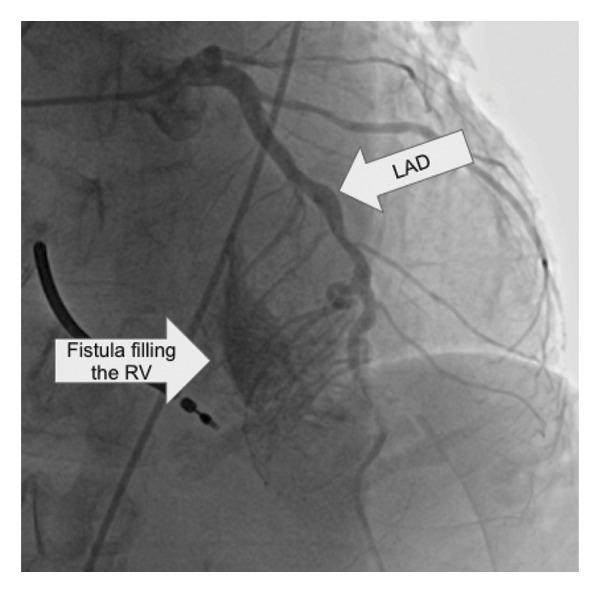
Left coronary angiogram demonstrating mid‐LAD cameral fistula to the right ventricle. The contrast started to fill the right ventricle.

**FIGURE 2:**
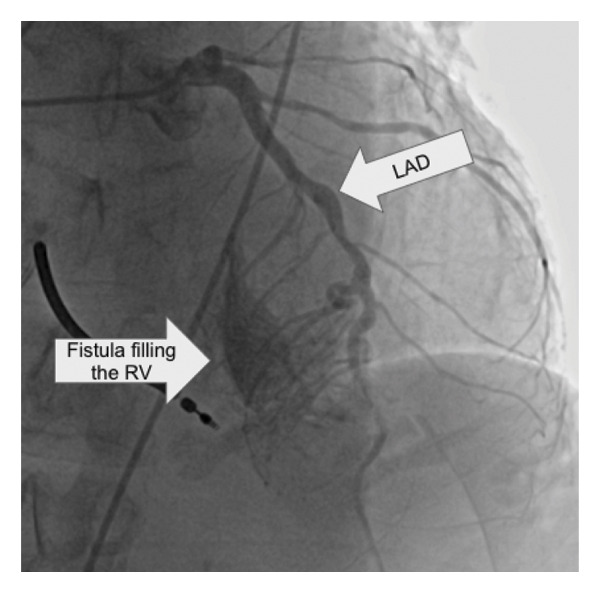
Coronary angiogram showing the filling of the right ventricle, suggesting a connection between the coronary flow and the right ventricle. The image illustration is a full RV‐gram as it was delineated with the white line.

A 73‐year‐old male with a past medical history of hypertension, hyperlipidemia, heart failure with reduced ejection fraction with an ICD in place, chronic hypothyroidism, nonrheumatic aortic valve insufficiency, and peripheral vascular disease presented with an elevated blood pressure of 161/117 mmHg, respiratory distress with bilateral wheezing, and accessory muscle use. On imaging, the patient’s chest X‐ray demonstrated pulmonary edema. Brain natriuretic peptide (BNP) was elevated to 3378 (normal range: 1–100 pg/mL), and troponin was elevated to 55 (normal range: 3–17 pg/mL). The electrocardiogram (ECG) showed a few runs of nonsustained ventricular tachycardia; his ejection fraction was around 20%–25% on transthoracic echocardiogram. Subsequently, the patient underwent cardiac catheterization, which showed a 99% distal stenosis of mid‐LAD with bridging collaterals from the RCA. A significant mid‐LAD coronary to RV fistula was noted on the angiogram (Figures 1 and 2). The patient showed symptomatic improvement postprocedure and was discharged on medications with outpatient follow‐up.

## 3. Discussion

Coronary artery fistulas are broadly classified into two categories, namely, (1) CCF, defined as fistulas between coronary arteries and cardiac chambers and (2) coronary arteriovenous malformations, which are abnormal connections between the coronary and systemic or pulmonary vasculature [5]. In our case, although the mid‐LAD coronary artery to RV fistula was an incidental finding and could be related to the patient’s acute heart failure, it provides a valuable opportunity to discuss CCF in detail. We discuss different types, incidence, prevalence, clinical presentations, and management approaches for these anomalies.

CCF can be either congenital or acquired vascular anomalies. The most common cause of CCF is abnormal embryogenesis, though trauma (e.g., stab injuries or gunshot wounds), spontaneous CCF, and iatrogenic causes, such as coronary angiography, pacemaker implantation, or endomyocardial biopsy, are also recognized triggers. Additionally, cardiac surgeries, including septal myomectomy, have been reported to cause these fistulas [4, 6, 7].

CCF are found in approximately 0.08%–0.4% of patients undergoing diagnostic coronary angiography. Around 90% of cameral fistulas communicate with the right‐sided chambers, while the remaining 10% drain into the left or both sides [8]. The most frequent drainage sites include, in decreasing order, the RV, RA, pulmonary artery, coronary sinus, LA, LV, and superior vena cava (SVC) [9]. One study identified five coronary artery‐to‐cardiac chamber fistulas, four of which originated from the RCA and one from the LAD. The RCA‐originated fistulas drained into the left atrium in two cases and into the LV in two others. The LAD‐originated fistula drained into the LV [10]. In contrast, our case demonstrated a fistula originating from the mid‐LAD draining into the RV, an uncommon presentation.

The clinical presentation of CCF depends on their hemodynamic significance, which is determined by the fistula’s connection’s size, the recipient chamber’s resistance, and the occurrence of myocardial ischemia [11]. Small fistulas often remain asymptomatic, but larger fistulas can result in a coronary “steal” phenomenon, leading to ischemia in the myocardial segment distal to the fistula. Complications of untreated, hemodynamically significant fistulas include cardiac failure, atrial fibrillation, bacterial endocarditis, thrombosis, embolism, rupture, sudden cardiac death, and premature atherosclerosis [5, 7, 12, 13]. Physical examination may reveal a continuous murmur, most often loudest over the precordium and typically peaking in mid‐to‐late diastole. In some patients with large shunts, signs of congestive heart failure or angina may be present, especially at the extremes of age [13].

The diagnosis of CCF is supported by various imaging and diagnostic techniques. Laboratory findings may reveal elevated cardiac enzymes and BNP in cases of heart failure. Chest radiographs are usually normal but may show chamber enlargement or pulmonary congestion with larger fistulas. ECGs are typically normal but may display signs of ischemia or chamber enlargement in more severe cases. Echocardiography, particularly transesophageal in adults and transthoracic in children, helps visualize the fistula and detect ischemia [4, 7, 13]. Most recently, Robu et al. demonstrated the role of TTE in detecting multiple moderate CCF originating from LAD and RCA and draining into the LV [14]. Similarly, in a case report of an RCA to RA fistula, initial TTE and TEE findings revealed dilated right heart cavities and systolodiastolic aliasing on color Doppler from the aorta to RA, along with visualizing multiple rounded hollow structures within the RA, highlighting the role of echocardiography in identifying the origin and distal entry sites of a CCF [15]. However, echocardiography remains limited in defining the detailed anatomy of a fistula. Advanced imaging methods, such as computed tomography (CT) and magnetic resonance imaging (MRI), provide detailed anatomical information. At the same time, coronary angiography remains the gold standard for determining fistula size and hemodynamic impact. Nuclear imaging may be used to evaluate myocardial ischemia, both pre‐ and postintervention [4, 7, 13].

Management of CCF depends on the size, origin, drainage, and symptoms. Most patients with CCF respond well to conservative management, with studies showing that 93% of patients do not require intervention and respond well to optimal medical management [16]. The response to conservative management depends primarily on fistula size, with small fistulas having the most favorable outcomes. The key determinant for intervention versus conservative management is fistula size. Small fistulas (diameter < 1 times the largest normal coronary vessel) typically close spontaneously over time and can be monitored without intervention [17]. Treatment is recommended for fistulas associated with significant left‐to‐right shunts, myocardial ischemia, arrhythmia, or ventricular dysfunction, as well as to prevent complications like heart failure or endocarditis as per the European Society of Cardiology and American Heart Association guidelines [18]. Surgical or catheter‐based closure is the primary treatment option. Surgery involves closing the fistula within the receiving chamber or vessel. If a large aneurysm is present, the feeding artery may be ligated within the aneurysm. Surgical outcomes are generally favorable, with a morbidity and mortality rate between 0% and 6%, and complete closure is achieved in over 95% of cases, though some residual fistulas may persist [7, 13]. Catheter‐based closure has become a safer alternative, utilizing embolization materials such as detachable balloons or micro coils. The choice of technique depends on factors like the anatomy of the fistula and the patient’s age. Successful occlusion requires precise catheter placement, especially in high flow or vessel tortuosity [19–21].

While coronary artery fistulas are rare, they represent an important clinical entity that can lead to significant morbidity if left untreated. This case, which involved an unusual mid LAD‐to‐RV fistula, highlights the diversity in fistula anatomy and underscores the importance of detailed imaging and careful clinical evaluation. Our case demonstrates the success of conservative management where aggressive symptom control using beta blockers resulted in clinically significant improvement. However, both surgical and catheter‐based interventions offer effective treatment options, with catheter‐based techniques emerging as a preferred approach due to their minimally invasive nature. Early diagnosis and treatment are crucial to preventing the potentially severe complications associated with large or symptomatic fistulas. Current ACC/AHA guidelines emphasize a heart team approach to evaluate the appropriateness of closure at centers with expertise in both percutaneous and surgical techniques [22].

## Funding

No funding was received for this manuscript.

## Consent

Written informed consent was obtained from the patient.

## Conflicts of Interest

The authors declare no conflicts of interest.

## Data Availability

The data that support the findings of this study are available on request from the corresponding author. The data are not publicly available due to privacy or ethical restrictions.
